# Combining O_2_ High Flow Nasal or Non-Invasive Ventilation with Cooperative Sedation to Avoid Intubation in Early Diffuse Severe Respiratory Distress Syndrome, Especially in Immunocompromised or COVID Patients?

**DOI:** 10.2478/jccm-2024-0035

**Published:** 2024-10-31

**Authors:** Fabrice Petitjeans, Dan Longrois, Marco Ghignone, Luc Quintin

**Affiliations:** Department of Anesthesia-Critical Care, Hôpital d’Instruction des Armées Desgenettes, Lyon, France; Bichat-Claude Bernard and Louis Mourier Hospitals, Assistance Publique-Hôpitaux de Paris, Paris Cité University, Paris, France; Department of Anesthesia-Critical Care, JF Kennedy North Hospital, W Palm Beach, Fl, USA

**Keywords:** ARDS, COVID, self-induced lung injury, spontaneous breathing, oxygen high flow nasal

## Abstract

This overview addresses the pathophysiology of the acute respiratory distress syndrome (ARDS; conventional vs. COVID), the use of oxygen high flow (HFN) vs. noninvasive ventilation (NIV; conventional vs. helmet) and a multi-modal approach to avoid endotracheal intubation (“intubation”): low normal temperature, cooperative sedation, normalized systemic and microcirculation, anti-inflammation, reduced lung water, upright position, lowered intra-abdominal pressure.

Increased ventilatory muscle activity (“respiratory drive”) is observed in early ARDS, at variance with ventilatory fatigue observed in decompensated chronic obstructive pulmonary disease (COPD). This increased drive leads to impending then overt ventilatory failure. Therefore, muscle relaxation presents little rationale and should be replaced by lowering the excessive respiratory drive, increased work of breathing, continued or increased labored breathing, self-induced lung injury (SILI), i.e. preserving spontaneous breathing. As CMV is a lifesaver in the setting of failure but does not heal the lung, side-effects of intubation, controlled mechanical ventilation (CMV), paralysis and deep sedation are to be avoided. Additionally, critical care resources shortage requires practice changes.

Therefore, NIV should be routine when addressing immune-compromised patients. The SARS-CoV2 pandemics extended this approach to most patients, which are immune-compromised: elderly, obese, diabetic, etc. The early COVID is a pulmonary vascular endothelial inflammatory disease requiring lower positive-end-expiratory pressure than the typical pulmonary alveolar epithelial inflammatory diffuse ARDS. This leads one to reassess a) the technique of NIV b) the sedation regimen facilitating continuous and extended NIV to avoid intubation. Autonomic, circulatory, respiratory, ventilatory physiology is hierarchized under HFN/NIV and cooperative sedation (dexmedetomidine, clonidine). A prospective randomized pilot trial, then a larger trial are required to ascertain our working hypotheses.

## Introduction

This article highlights the pathophysiology of classical vs. COVID-acute respiratory distress syndrome (ARDS), and the use of O_2_ high flow nasal (HFN) and very high flow nasal (VHFN>70 L.min^−1^) and inspiratory assistance (pressure support: PS) to avoid endotracheal intubation (“intubation”). This is a follow up of a manuscript devoted to early weaning of invasive ventilation [9]. In the setting of COVID-ARDS, ~41% of the patients received HFN or non-invasive ventilation (NIV) or continuous positive airway pressure (CPAP) [10], but only ~20% of the patients receive analgesics or sedatives [11]. Indeed, sedation is believed to cause respiratory depression and conceal ventilatory failure (“failure”) i.e. the clinical sign to escalate to more invasive therapy. By contrast, alpha-2 agonists (“cooperative sedation”, rousable sedation: dexmedetomidine, clonidine, etc.) are now considered as first-line sedatives in the critical care unit (CCU) [12,13,14,15,16,17]: dexmedetomidine eases NIV [18] and halves the occurrence of endotracheal intubation (“intubation”) [19].

CMV is lifesaving [20] when impending or overt ventilatory failure is ominous. Nevertheless, CMV “*(in and of itself) does not produce lung healing*” [21]. In multiple-organ failure patients under conventional sedation, CMV is associated with death ranging from 16 to 88 % [22,23,24] (discussion: [25]). Current management [26] is associated with circulatory disturbances, ventilator-associated pneumonia, excessive sedation, delirium, muscle weakness, immunoparalysis, etc. Thus, NIV is the first line tool in the setting of immunodeficiency [27, 28] or upon massive influx of elderly patients with baseline chronic inflammation and comorbidities.

Continued or intensified labored breathing (“labored breathing”) [29] leads to impending, then overt failure, additional lung injury (inflammation; self-induced lung injury: SILI [5, 30]; ventilator-induced injury: VILI). Thus, delayed intubation and ventilatory assistance may lead to overt failure, gasping, cardiac arrest and death [30,31,32,33,34].

A multimodal approach [9, 35,36,37,38,39,40] (“analytical management” [37,38,39,40,41]) hierarchizes the pathophysiology of the autonomic nervous system, the respiratory generator [42,43,44], the vasomotor center [45], the chest wall and lung mechanics [6, 8], circulation [46], kidney and metabolism. The interval between admission and intubation gives one the opportunity to address labored breathing [29], reduce the inspiratory effort (large negative esophageal pressure change), normalize the work of breathing (WOB), reverse failure, break-up SILI [40] and bypasses intubation. Our hypothesis is: cooperative sedation extends the tolerance to HFN or NIV and buys time for a multimodal approach [35] to normalize the respiratory drive. As this multimodal approach bears many research questions, they are delineated in the appendix.

## Pathophysiology

### Acute respiratory distress syndrome: pathophysiology

Very schematically, early diffuse ARDS entails alveolar *epithelial* dysfunction. By contrast, early COVID-ARDS entails pulmonary vascular *endothelial* dysfunction [6, 8, 47, 48].

ARDS is a broad entity characterized by severe dyspnea, hyperpnea, tachypnea, hypoxemia, decreased lung compliance (“compliance”), alveolar infiltrates [49], redefined as PaO_2_/FiO_2_=P/F<300/200/100 with positive end-expiratory pressure (PEEP)=5 cm H_2_O after intubation, bilateral opacities without volume overload or cardiac failure [23]. This extends to non-intubated patients [50]. These criteria are not perfect [51]. Using PEEP= 10 cm H_2_O, FiO_2_=1 leads to underestimate ARDS [52]. FiO_2_=1 at low PEEP de-recruits alveoli and lowers P/F (196 to 153) [53]. As ARDS entails a spectrum of diseases [54] and several clinical presentations (“phenotype”), a CT scan individualizes management:

#### 1. Typical ARDS

Typical ARDS [6] comprises two entities [55]: early diffuse ARDS entails alveolar epithelial dysfunction, unstable alveoli, fluid-filled alveoli (non-cardiogenic pulmonary edema), bilateral infiltrates that ultimately coalesce into compressive atelectasis. A direct, proportional, relationship exists between the amount of non-aerated tissue and lowered compliance [6]. Typical ARDS is addressed with high PEEP [56], except in the setting of “focal “ ARDS [55].

– “focal” ARDS entails extra-pulmonary ARDS, loss of hypoxic vasoconstriction, high compliance and low inflection point on the inspiratory pressure-volume (P-V) curve (≤5 cm H_2_O) [55], and is addressed with low PEEP.
– “diffuse” ARDS entails pulmonary ARDS, high dead space and PaCO_2_ [57, 58], low compliance [59] (*a “baby” lung is small but not “stiff”* [60]), high inflection point (>7 cm H_2_O), high mortality and is addressed with high PEEP [56]. High inflection point is related to low end-expiratory volume, low functional residual capacity (FRC) [55] and low PaO_2_.

#### 2. COVID-ARDS

Early COVID-ARDS entails pulmonary vascular endothelial dysfunction [47, 48], pulmonary vascular abnormalities [6], loss of hypoxic vasoconstriction with hyperperfusion of non-aerated, gasless tissue at variance with areas of no-perfusion and normal aeration [6], micro- and macroemboli [47, 48, 61], well aerated lung volume [62], high compliance and low driving pressure (DP) [63]. Intrapulmonary shunt (“shunt”) is perfusion of non-aerated alveoli (low or zero VA/Q [4]). The implication is that a high shunt fraction goes to gasless tissue [62]. Micro-emboli prevent recruited alveoli to participate in gas exchange. Venous admixture is intrapulmonary shunt+VA/Q mismatch [8]. In COVID-ARDS, VA/Q mismatch is more important than shunt i.e., predominantly low perfusion of ventilated alveoli. By contrast, in typical ARDS, shunt is more important than VA/Q mismatch i.e., adequate perfusion of nonventilated alveoli [8] (COVID-ARDS: high VA/Q and dead space; diffuse typical ARDS: low VA/Q) [63]). In COVID-ARDS, profound hypoxemia [48] occurs when compared to typical ARDS with same compliance. Typical ARDS presents with a higher P/F for the same compliance [6]). Recruitment is highly variable [63]. In the COVID-ARDS setting, low Vt results in increased dead space, reabsorption atelectasis, hypoventilation, hypercarbia, high hypercapnic drive and high sedative requirement. Low Vt-high PEEP conventionally proposed in typical ARDS appears of modest benefit in COVID-ARDS [6, 62].

*Hypoxic vasoconstriction* is relevant given the vascular disease. In pig lung injury, PS ventilation is associated with a redistribution of blood flow toward non-dependent better aerated lung without inducing recruitment. Increased aeration and improved hypoxic vasoconstriction occur in dependent regions [64]. Furthermore, alpha-2 agonists improve hypoxic vasoconstriction [65,66,67].

The mechanisms observed in early ARDS progress toward fibrosis more rapidly in COVID-ARDS compared to typical ARDS. Consequently, starting from admission, the intensivist is essentially racing against time, contending with ventilatory failure on one front and the rapid progression towards fibrosis on the other.

### Ventilatory failure: impending vs. overt

**Fig. 1.**
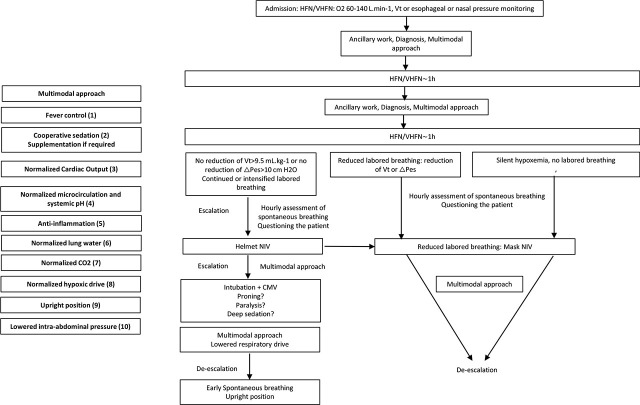
**From non-invasive to invasive ventilatory assistance in the setting of severe ARDS**. The clinical signs of ventilatory failure are: discomfort, intolerance to device, mental deterioration, diaphoresis, dyspnea (hyperpnea> tachypnea), inspiratory effort [use of accessory muscles, phasic activation of the sternomastoid muscle (palpation of the sterno-mastoid muscle as an index of drive in ARDS [69]), tracheal tug [69], thoraco-abdominal swing, suprasternal notch retraction (index of large negative esophageal pressure change), intercostal recession [69], nasal flaring, gasping [70]], copious respiratory secretions [71], airway bleeding, circulatory instability, electrocardiographic changes, P/F trend. An index of drive, airway occlusion pressure (P0.1), is set to 0.5 ms in the spontaneous breathing setting (P0.5) [125] and used as such. *Isolated “silent hypoxemia” without signs of labored breathing as the principal symptom*: HFN/VHFN is the logical therapy. A multimodal approach complements HFN/VHFN to allow for an extended period of optimization and observation. *Labored breathing as the principal symptom*: continued or intensified labored breathing should be interrupted to avoid transitioning from impending to overt failure and arrest. HFN/VHFN allows one to simultaneously buy time, observe, carry on the ancillary work (insertion of lines, chest X Ray, ECG, CT scan, bronchoscopy, pleural-pulmonary-cardiac ultrasounds, etc.) and addressing hypoxemia. Repeated assessments of the tidal volume (under mask NIV) and other signs of ventilatory failure or nasal/esophageal pressure [70] changes will allow one to assess improvement or deterioration within 2 h (NIV failure vs success respectively: Vt: 9.6–12 vs. 7.6–10.2 mL.kg^−1^ with NIV set to Vt=6–8 mL.kg^−1^ [72]; NIV success: reduction in esophageal pressure change≥10 cm H_2_O [2]; nasal pressure change mirrors esophageal pressure change [70]). Criteria for escalation from HFN to NIV are P/F<100, and/or RR>25 bpm, and/or respiratory distress and dyspnea despite HFN>60 L.min^−1^ [70]. Absence of improvement or deterioration within 2 h suggests switching to helmet NIV to achieve higher PEEP, restore a fluid-like lung behavior and reduced work of breathing. Absence of improvement or deterioration implies running through a multimodal approach again, looking for sepsis, coronary artery, delirium tremens, etc., then escalating up to intubation+CMV and avoiding overt failure. NIV is set to avoid dyssynchrony: low inspiratory trigger, high pressurization time, lowest expiratory trigger. Helmet NIV requires faster pressurization time≤50 ms, cycling off=30% of peak inspiratory flow, higher PS level (+33–50%) and PEEP. High inspiratory assistance should not sum up with negative esophageal pressure change to avoid high transpulmonary pressure and further inflammation. HFN or NIV allows one to buy time and combine physiological tools (circulatory, respiratory, ventilatory, autonomic) within a multimodal approach. The check list is *(Vt, RR)=f(temperature, agitation, cardiac output, microcirculation-arterial lactate, inflammation, lung water-diuresis, systemic pH, PaCO_2_, PaO_2_):*
1)*fever control* [156, 157]: 36<θ<35°C, i.e. first [paracetamol, wet sheet+fan or BairHugger^®^] then alpha-2 agonist (“*no bolus, start slow-go slow, fill them up-then open them up*”; dexmedetomidine or clonidine up to 1.5 or 2 µg.kg^−1^.h^−1^, respectively). Alpha-2 agonists develop favorable effects slowly (≥3 h) if a slow administration is used to avoid bradycardia or hypotension, *after* iterative echocardiographic assessment and passive leg raising.2)*agitation* [167] addressed to stringent quietness (−2<RASS<0; cooperative sedation: alpha-2 agonist as first-line sedative [15]; “breakthrough”: haloperidol 2.5–10 mg bolus or 5 mg bolus up to 4 administrations; supplementation: infusion up to 50 mg/day).3)*normalized cardiac output* [4, 46]: *iterative* echocardiography coupled with volume, vasopressors, inotropes, pulmonary vasodilators.4)*normalized microcirculation* and pH (systemic and regional): the alpha-2 agonist normalizes the sympathetic vascular activity, revascularizes the microcirculation, normalizes the local pH and arterial lactate and inflammation linked to acidosis.5)*anti-inflammation* (source control; alveolar antiinflammation: adequate PEEP to suppress atelectrauma; systemic indirect antiinflammation i.e., alpha-2 agonist, steroids).6)*reduced lung water*: volume loading before PEEP and administration of alpha-2 agonists then according to clinical signs, lowered PCWP [241] or iterative echocardiography. Increased CO or BP upon passive leg raising does *not* necessarily imply further volume loading. Only peripheral perfusion dictates volume load: mottling, capillary refill time, diuresis, lactate, pH, CO_2_ gap, mixed venous saturation. The alpha-2 agonist produces anti-ADH effect, diuresis and kaliuresis.7)*normalized CO_2_*: lowered activity of the respiratory generator and inspiratory muscles through fever control (36<θ<35°C), microcirculation and pH with an alpha-2 agonist. PS level is as necessary to suppress the additional work of breathing caused by the valves and tubings (3–7 cm H_2_O) [116].8)*normalized hypoxic drive* [78]: Oxygen therapy is the first line upon admission. a) FiO_2_=1 as briefly as possible (absorption atelectasis, toxicity) lowered step by step to 0.4, without lowering the flow, i.e. keeping PEEP on. Normalization of systemic CO_2_ and pH are key before normalizing the hypoxic drive. b) PEEP according to the disease : focal ARDS : 5 cm H_2_O; COVID-ARDS: 8–10 cm H_2_O; diffuse ARDS: 16 cm H_2_O. An esophageal balloon individualizes PEEP as early as possible. Leaks in the setting of NIV limit the ability to use very high PEEP.9)*upright position* [7]: reverse Trendelenburg, 60° head up, 45° leg down. Upright position makes compression stockings or military antishock trouser sometimes useful.10)*lowered intra-abdominal pressure*: gastric and bladder decompression, increased colonic motility (mild laxative). Abbreviations: HFN: O_2_ high flow nasal; VHFN: very high flow nasal; NIV: non-invasive ventilation; CMV: controlled mandatory ventilation; PEEP: positive end-expiratory pressure; PS: pressure support, inspiratory assistance.

Upon admission, the clinical presentation involves silent hypoxemia *or* ventilatory muscle dysfunction (“muscle dysfunction”) with labored breathing [10] (Figure 1). This dictates the immediate management whether it be HFN or helmet NIV, respectively.

*Semeiology*: If the use of HFN/VHFN/PS does not quickly alleviate labored breathing, impending failure is an indication for intubation+CMV. There is no definitive index that mandates intubation, but rather continued observation of ongoing or worsening failure. Clinical signs to look for are discomfort, intolerance to the device, mental deterioration, diaphoresis, dyspnea (with hyperpnea being more relevant than tachypnea), increased inspiratory effort, phasic activation of the sternomastoid muscle (palpation of the sternomastoid muscle allows for assessment of the drive in decompensated chronic obstructive pulmonary disease (COPD) [68] and ARDS [69]), use of accessory muscles, tracheal tug [69], thoraco-abdominal swing, suprasternal notch retraction (an index of large negative esophageal pressure swing), intercostal recession [69], nasal flaring, gasping for air [70], copious respiratory secretions [71], airway bleeding, circulatory instability, electrocardiographic changes, trends in P/F ratio.

*Criteria for intubation* are primarily based on the clinical evolution. Labored breathing [29], overt failure [21], continuing hyperpnea (Vt>9.6–12 mL.kg^−1^ [72]) or absence of reduction of esophageal change<10 cm H_2_O [2] are ominous signs. RR>30–35 min^−1^, SaO_2_<88% are only contributive. Tachypnea is a response to lung inflammation but does not alone justify intubation [21]. In the setting of HFN, even minimal tachycardia is a sign of failure (intubation: HR=108±19; not intubated: 104±19 beats per min [33]).

#### 1. Silent hypoxemia

Hypoxemia results from reduced O_2_ diffusion (typical ARDS) or inadequate alveolar perfusion (COVID-ARDS: micro- or macroemboli [48]) and is not necessarily accompanied by muscle dysfunction and signs of ventilatory failure, e.g., during “silent hypoxemia” [73,74,75]. Isolated hypoxemia without labored breathing is addressed with HFN/VHFN. Nevertheless, prolonged silent hypoxemia may lead to clinical deterioration, continued labored breathing, and eventually intubation.

#### 2. Clinical evolution

The present opinion regarding *clinical* evolution aligns with recent guidelines on the use of HFN/NIV in the context of COVID (Table 1 [76]). One notable difference worth discussing is the emphasis on SaO_2_>92% in the consensus paper [76], while others differentiate requirements based on P/F [23, 50]. Some experts [20, 21] stress the importance of observing the evolution of clinical signs, such as hyperpnea rather than tachypnea, over relying on oxygenation parameters. Intubation decision is not based upon SaO_2_ or PaO_2_ values [20, 21], or segregation with P/F [23, 50]. The indices help in identifying NIV failure within 2 h of treatment initiation [2, 72, 77] (HFN: ROX index: NIV; HACOR; table 1 in [10]). Subsequent observation is performed on an hourly basis. (Figure 1). Silent hypoxemia is a contributing factor to this approach. Additionally, hypoxemia acts as a short-acting, unsustained stimulus, known as “hypoxic ventilatory decline,” which primarily heightens the response to acidosis or hypercapnia [9, 74]. For instance, even when SaO_2_ is ≤70%, a PaCO_2_ of approximately 32 mm Hg (end-tidal <29 mm Hg) can prevent the hypoxic response [74]. Therefore, the conventional threshold (PaO_2_ > 55–60 mm Hg, with SaO_2_ > 92%) provides only a rough estimate, indicating merely the flat portion of the O_2_ dissociation curve. Nevertheless, being outside this flat portion does not necessarily mandates intubation. The initial line of therapy focuses on restoring oxygenation and normalizing the hypoxic drive [78], which may not immediately necessitate intubation but instead requires close observation looking for worsening failure and a multimodal approach.

**Table 1.** Criteria for non-invasive ventilatory failure [76]

Absence of improvement or worsening of clinical signs observed on admission, including oxygenation data and increased respiratory rate Appearance of signs of ventilatory muscle fatigue or use of accessory muscles Presence of acidosis, both respiratory and metabolic Inability to properly clear respiratory secretions Signs of circulatory instability, including hyperlactatemia Deterioration of consciousness or presence of seizures Intolerance to device, especially mask wearers

Muscle dysfunction involves overly active muscles. In contrast to the acute over chronic fatigue seen in COPD, muscle function in ARDS is typically intact at baseline, i.e. prior the onset of ARDS. However, muscle failure can occur due to various factors, including a) septic dysfunction [79], b) acute cardiac failure leading to exhaustion and death [80], and c) prolonged evolution (as mentioned above).

In early ARDS, a high inspiratory activity (“respiratory drive”, “drive”, “neural demand” [42, 43]) impinges upon intact muscles. A high muscular activity of intact muscles requires transpulmonary pressure to be addressed specifically (low inspiratory assistance, low pressure support: PS using upfront helmet NIV). This contrasts with acute over chronic fatigue of muscles observed in decompensated chronic COPD with reduced CO_2_ excretion: in the setting of COPD, unloading the muscles is necessary for hours or days with high PS to overcome fatigue and decompensation [68]. By contrast, high PS is inappropriate for ARDS.

#### 3. Respiratory drive

Respiratory and ventilatory physiology refer to brain stem processes vs. lung and chest wall function, respectively. Located in the lower brain stem, apposed to the vasomotor center, the respiratory generator (“generator”) controls the respiratory rhythm and phrenic activity and integrates the myriads of factors leading to the drive and the activation of the ventilatory muscles: *(Vt, RR)=f(temperature, agitation, cardiac output, microcirculation, inflammation, lung water-diuresis, systemic pH, PaCO_2_, PaO_2_;* Equation 1, to be used as a check list). ARDS patients present with a high drive, ventilatory muscular activity, inspiratory peak flow (“air hunger”) [2, 72, 81, 82], transpulmonary pressure, inflammation, labored breathing and impending failure. The higher drive present in COVID-ARDS patients led away from light sedation and spontaneous breathing (SB) [83,84,85], back to deep sedation, paralysis, protective ventilation and proning. As emphasized early April 2020 physiology was at loss in a bewildered world (francais.medscape.com/voirarticle/3605845?=null&icd=login_success_email_match_fpf&form=fpf). Dissecting and normalizing the myriads of factors [35, 74] involved in the genesis of hyperpnea and tachypnea allows one to lower the drive immediately following admission (“multimodal approach” [35]). Normalized drive rests on a functional generator at variance with the suppressed activity of the generator and suppressed drive caused by general anesthesia+paralysis.

#### 4. PEEP

As oxygenation is not the key issue anymore in ARDS [21, 35], the rationale for using high PEEP does not rest on oxygenation. Poor oxygenation (P/F<150) is *unassociated* with the increased inspiratory activity [2] but with inflammation [86].

In the setting of diffuse alveolar damage, solid-like behavior leading to pendelluft, increased spontaneous ventilatory effort [35, 87], atelectrauma, WOB and SILI are to be avoided. High PEEP prevents cyclic collapse of the bronchiolar tree [88] or of alveoli (atelectrauma) [89], thus suppresses the mechanical inflammation (SILI or VILI). As observed during the first breath after birth (−70 cm H_2_O [90]), the first inflation of a kid’s balloon requires very high transmural (transalveolar) pressure; further inflation requires minimal incremental pressure. Once inflated by adequate PEEP, the “baby lung” in adult ARDS operates on the highest slope of the *expiratory* [91] P-V curve (highest compliance). The lung switches from solid- to fluid-like behavior [2], with a reduction in esophageal pressure changes and DP. The low PEEP achieved with HFN/NIV cannot recruit all atelectatic, non-aerated, areas. The objective is only to improve low VA/Q areas [92, 93], at variance with fully reopening the lung [94, 95] and correcting entirely the intrapulmonary shunt. Such a minimalist objective requires much lower PEEP levels. If so, “protective” ventilation is not protective because of low Vt, but because of PEEP and reduced solid-like behavior, WOB, pendelluft [87] and atelectrauma. Recruitment increases resting volume and FRC, lowers DP and decreases lung deformation (strain [5], Vt/end-expiratory lung volume ratio [6]). By contrast, low PS, Vt and transpulmonary pressure minimizes stress [5, 6]. In addition, under SB, the active diaphragm keeps the alveoli open during a longer expiratory interval [96], synergistically with PEEP.

The low PEEP achieved with HFN/VHFN/NIV may suit the relatively high compliance and low PEEP requirements observed in the setting of early COVID-ARDS [61, 63] and focal ARDS. PEEP is set as a function of the considered disease, using various techniques a) immediately following admission, a “*one size fits all*” approach uses the ARDS network table [97, 98]. Evidently, leaks observed in the setting of mask NIV would not allow setting the highest PEEP levels. This bears little consequences in the setting of focal ARDS or COVID-ARDS as lower PEEP levels are required when compared to diffuse ARDS. b) given a CT scan, rules of thumb are useful: “focal” ARDS: ~5 cm H_2_O; diffuse ARDS: ~10 cm H_2_O [55]; mild vs. severe: 5–10 vs. 15–20 cm H_2_O [98]; COVID-ARDS: 8–10 cm H_2_O [48] c) titrated to respiratory compliance [8] (COVID: avoid overdistension, increased dead space, hypercarbia and heightened drive; typical ARDS: recruitment of perfused units: low VA/Q, and increased compliance d) as soon as possible, an esophageal balloon individualizes PEEP [99, 100] in SB patients [2, 70]. e) impedance tomography or lung echography combined to arterial and venous gases and echocardiography are another option.

### Limits of controlled mechanical vs. spontaneous ventilation

#### 1. Limits of controlled mechanical ventilation

ARDS is managed [26] using intubation, general anesthesia [101] (GA renamed “deep sedation” [102], analgesia-sedation), paralysis [103], proning [22] and low DP [104]. Nevertheless, this is *not* a treatment for ventilatory failure [21]: CMV only buys time [49] for self-healing [21, 105] of the alveolus or of the capillary. Many issues are unsettled:

- despite remarkable results [103], paralysis should be used sparingly, e.g., high drive [106].
- deep sedation is associated with ventilator-to-patient dyssynchrony [107] and death [85] in ARDS patients [108].
- no comparison of SB vs. paralysis [109] has been published. SB with airway pressure release ventilation [110, 111] is not discussed. Three issues are to be considered:
  - no trial addresses the various Vt in the setting of ARDS (2, 4, 6, etc. mL.kg^−1^; appendix). The only evidence is the retrospective linear association between improved outcome and lowered DP<14 cm H_2_O [104].
  - proning: That many humans sleep in the prone position is not an argument for awake proning in early COVID-ARDS: humans move freely from supine to prone and back during sleep, at variance with imposed prolonged proning in a CCU environment with discomfort.

The excellent epidemiological result [22] is methodologically weak. First, the results are not segregated between P/F<100 vs. <150, mixing severe and moderate ARDS. Second, no comparison exists between supine vs. prone vs. lateral+prone+lateral positioning; *multiple* repositioning is presumably the key to address compressive atelectasis, but not necessarily proning itself. Third, P/F returns towards baseline after turning supine, with no comparison to supine group (figure S2 [22]). The *cause* of ARDS is unaltered by proning; a rescue therapy causes no miracle. Simply the number of patients requiring proning progressively decreases, improved by multiple repositioning.

Mechanically, collapse is a function of the hydrostatic pressure imposed on the alveolus (i.e. the weight of the heart on the left lung). Thus, proning opens more non-dependent dorsal zones than it collapses in dependent sternal regions [112]. Indeed, proning leads often to a small improvement in PaO_2_ [62], due to better VA/Q matching in vaso-dysregulated tissue [6] or perfusion redistribution in response to pressure or gravity [62] but not to alveolar recruitment. Given high compliance, minimal P/F improvement linked to CCU proning presents minimal interest in the setting of early COVID-ARDS, imposing on limited staff resources ([6] “responders”: P/F increase >20 mm Hg in 75% of the patients; [62, 113]). In intubated patients, proning vs. upright position increases P/F to a similar extent in patients with the lowest P/F (moderate and severe ARDS, panel B, *Appendix* vs. table S8 [7, 22]). Proning used in the setting of intubated paralyzed patients [22] was extended to awake non-intubated patients in the setting of early COVID-ARDS. Given these limitations, proning in awake non-intubated patients may avoid intubation [8].

  iii. SB: Severe ARDS in *single*-organ failure patients managed with early SB under cooperative sedation carries a ~8.5% mortality in COVID-ARDS [39].
  iv. absence of sedation: Passive hyperventilation below the apnea threshold *without* sedation carries a low mortality in COVID-ARDS [114].
d. the absence of a prone vs. upright position trial (reverse Trendelenburg, head-up +60°, legs down: −45°) [7].

These weaknesses leave recommendations [26] with shaky foundation [115]: “*loss of muscle tone…. caused by muscle relaxants, anesthetics, and sedatives, and the use of high oxygen concentration in inspired gas are the prerequisites to produce atelectasis in…. healthy subject during anesthesia. This…. common treatment in ARDS… adds to the collapse and consolidation caused by the disease itself*”.

#### 2. Limits of spontaneous breathing

- CPAP vs. inspiratory assistance: HFN provides CPAP and increased end-expiratory volume *without* inspiratory assistance. Thus, it does *not* unload the inspiratory muscles. If a high drive is not normalized early, and given the load imposed by the valves and tubing [116], this absence of unloading may progressively cause acute fatigue of intact muscles, requiring switching to PS or CMV to prevent progression to failure.
- High vs. low inspiratory assistance: The high inspiratory effort, and Vt, is influenced by inflammation and drive but independent of the level of inspiratory assistance (PS level) [72, 117]. A high inspiratory effort manifests as hyperpnea, hypocapnia, a large inspiratory esophageal pressure drop (∆Pes=26–40 cm H_2_O) and low dynamic compliance (Vte/∆P_L_) [2]. Excessive inspiratory assistance further amplifies the inspiratory esophageal pressure change, the transpulmonary pressure [81, 118], Vt, atelectrauma, and inflammation. Therefore, inspiratory assistance is required only to alleviate the WOB caused by the ventilatory apparatus rather than to unload the muscles (valves, tubings; PS=3–5 cm H_2_O [116]). More, inspiratory assistance cannot alleviate solid-like behavior, atelectrauma and mechanical inflammation. Adequate PEEP can achieve this when the lung is at its optimal compliance. Setting PS to achieve a Vt=7–10 mL.kg^−1^ [33] will completely unload the ventilatory muscles but may be detrimental because the baby lung does not tolerate such high Vt [119]. Rather, esophageal [2] or nasal [70] pressure changes should be limited. Therefore, an uncontrollable drive leading to labored breathing and increased WOB does not necessitate increased PS but rather a reduction in drive, with early initiation of helmet NIV [82, 120]. Failed NIV is defined as the absence of reduction in ∆Pes<10 cm H_2_O within 2h (reduced dyspnea and hyperpnea i.e., success: ∆Pes=8–15; failure: 30–36 cm H_2_O) [2]. Accordingly, increased Vt>9.6–12 mL.kg^−1^ is the hallmark of early NIV failure [72]. NIV failure is associated with death either because of uncontrollable drive in a very sick patient [20], or a too high PS level. By contrast, successful NIV require close observation with early escalation only if continued labored breathing persists: HFN, VHFN, mask NIV, helmet NIV (Figure 1).

## Oxygen high flow nasal and non-invasive ventilation

### Oxygen high flow nasal

Classical [121] or updated [122, 123] Optiflow™ help normalizing labored breathing while simultaneously addressing the ancillary work (figure 1) and a multimodal approach [35]. In the setting of early ARDS, HFN takes precedence over NIV [124], with certain caveats [82, 120]. HFN increases CO_2_ wash-out and dynamic compliance, comfort, oxygenation [125] and clearance of secretions [10]. HFN reduces inspiratory effort, CO_2_ production and RR due to a resistive effect and prolonged expiration. The degree of improvement correlates with the flow rate and PEEP, leading to increased FRC, restored fluid-like behavior, and decreased inspiratory WOB. For instance, administering HFN at 50 L.min^−1^ to patients in septic shock diminish the respiratory drive (P 0.5) and esophageal pressure change [125].

With HFN, O_2_ flow up to 60–80 L.min^−1^ is achieved through conventional Optiflow or a ventilator. Modified Optiflow can administer up to 120 L.min^−1^ [123]: two blenders into one nasal prong convey a very high flow (VHFN; 1.5 mL.kg^−1^ [123]). In healthy volunteers, the mean airway pressure ranges from ~3 to ~12 cm H_2_O, generating PEEP (35 L.min^−1^: range: 1.5–5.3 cm H_2_O; 100 L.min^−1^: range: 7.3–16.2 cm H_2_O [121, 122]). In the setting of early focal and COVID-ARDS, this may allow enough recruitment to avoid intubation when silent hypoxemia without labored breathing is the principal derangement. However, VHFN appears poorly tolerated after 20 min [123]. The reason is unclear : poor humidification? high expiratory resistance and expiratory WOB [123]? This leads to the combination of discontinuous NIV and discontinuous HFN, making the technique complex and possibly inadequate to avoid intubation.

In the setting of acute failure, high inspiratory peak flow leads to room air entrainment under HFN. Exercise generates a peak inspiratory flow up to 255 L.min^−1^ [126, 127] and mimics the peak flow observed during acute failure [127]. A challenge is to match such a high peak inspiratory flow. Simple tools minimize air entrainment, either alone or combined:

– A simple surgical mask applied over the mouth in addition to HFN 60 L.min^−1^ decreases the RR (28 to 26 breaths per min: bpm), increases the PaO_2_ (59 to 79 mm Hg) and P/F (83 to 111) [128]. Adding a “double trunk” mask without adding O_2_ to HFN=40–60 L.min^−1^ increases PaO_2_ (63 to 88 mmHg in 11 responders out of 15 patients) [129].
– in healthy volunteers, HFN 50 L.min^−1^ within a standard helmet achieves stable high PEEP=8 cm H_2_O and increases CO_2_ washout (PetCO_2_=33 mm Hg) [130].
– in addition to the nasal prong, insertion of up to 2 prongs through the mouth can achieve O_2_ flow~120–180 L.min^−1^. In our experience, two classical Opti-flow prongs, oral and nasal, achieves O_2_~120–140 L.min^−1^ and high SaO_2_ (Quintin, unpublished data).
– cooperative sedation (above: 2<RASS<0) evokes indifference to CCU stimuli and lowered VO_2_, enhancing tolerance to continuous HFN/NIV, noise, humidification, and nasal prong(s) for days.

### Non-Invasive ventilation

Labored breathing and fatigue lead to NIV, which is a consequence of either continued or increased drive or to the absence of any inspiratory assistance with HFN/VHFN. Criteria for escalation to NIV are P/F<100, and/or RR>25 bpm, and/or ventilatory distress and dyspnea despite HFN>60 L.min^−1^ [70].

*Ventilator-to-patient dyssynchrony*: Using NIV, the key issue is to adapt the ventilator to the patient, not the opposite [132]. First, inspiratory effort occurs during early inspiration, especially when high inspiratory activity occurs and low flow settings are used [133]. Patient-ventilator dyssynchrony occurs only if the ventilator’s inspiratory assistance is suddenly lost during continued inspiratory muscle contraction [134]: the more intense the drive, the higher the flow requirement [135]. Second, the ventilator’s inspiratory cycle should stop immediately before the beginning of the patient’s expiratory effort [133].

#### 1. Mask NIV

To our surprise, with a tightly adjusted mask, Drager ventilators (Evita XL, Infinity V500) combined to cooperative sedation allows for achieving PEEP up to 20 cm H_2_O with minimal leaks, for days [136]. Despite leaks and tolerance issues, since the pathophysiology of ARDS differs only in the amplitude of the dysfunction in intubated vs. non-intubated patients and the literature is limited, parameters set under invasive ventilation are used [9, 36, 37, 40]:

- *PEEP* is set on a patient-per-patient basis given the heterogeneity of ARDS. Leaks lower PEEP; however, the patients treated with NIV are less severe or present to the CCU earlier in the evolution of their ARDS. An esophageal balloon inserted as early as possible allows for observing reduced esophageal pressure change (∆Pes<10 cm H_2_O [2]) and improved labored breathing. In some patients, NIV appears successful within minutes when high PEEP combines with low PS [136], possibly restoring fluid-like behavior.
- *PS*: Under PS, plateau pressure (Pplat) is measured during a brief inspiratory hold in intubated patients [137]. NIV was initially used in the setting of acute decompensation of COPD with muscular fatigue, and thus requiring high PS amplitude. As the patient population has switched from COPD [68] to ARDS and SILI [5], inspiratory assistance is lower:
  - PS=5, PEEP=5–15 cm H_2_O, high Vt (500–600 mL) resulting in improved dyspnea in the setting of early ARDS following acquired immunodeficiency syndrome [138].
  - PS=7 cm H_2_O, PEEP<10 cm H_2_O to minimize leaks [72]. The Vt was ~8–9 in the success group vs. 11–12 mL.kg^−1^ in the failure group. In contrast, late ARDS is characterized by low Vt (rapid shallow breathing: ~4.2 mL.kg^−1^ [139]).
  - In our experience, with a normalized WOB, the “*Smartcare*”™ software [140] is highly efficient in reducing PS (Drager Evita 4XL or Infinity V500 with Smartcare) [141]. Smartcare reduces PS from a preset level ~6–8 cm H_2_O to a final level ~3–5 cm H_2_O. The inspiratory WOB is almost entirely suppressed with no phasic activation of the sternomastoid muscle and no sternal notch retraction (high PEEP-low PS termed “inverted settings” [36]). Indeed, a meta-analysis suggests that high PEEP-low PS lowers intubation rate from 43 to 25 % (PEEP=8±2 cm H_2_O, PS=7±2 cm H_2_O) [142].
  - The reduction in esophageal pressure changes observed in the NIV success group is associated with the following initial settings: PEEP~10 cm H_2_O and P~10 cm H_2_O adjusted to achieve Vt<9.5 mL.kg^−1^ [2]. After 2 h, PS was lowered (~11 cm H_2_O to ~9 cm H_2_O) in the *failure* group to decrease the Vt [143]. Nevertheless, in contrast with our proposed high PEEP-low PS, the observed Vt was ~11 mL.kg^−1^ regardless of success or failure (table 2 [2]). When compared to failure, success is associated with lower esophageal pressure change, higher P~17 cm [143] and similar Vt [2].
  - in the setting of ARDS, a low inspiratory assistance (PS=6 cm H_2_O) was used to confirm high Vt independent of PS level [144].
- *Inspiratory trigger* at the lowest level: surprisingly, under cooperative sedation, delineated below, and normalized drive, no asynchrony is observed (monotonous breathing, no breath stacking, no double triggering).
- *Slope of pressure ramp*: The highest possible pressurization time generates a short inspiratory rise time and leads to the shortest and highest inspiratory peak flow [145]. This minimizes inspiratory effort, esophageal pressure change [146], pendelluft, extracapillary fluid filtration, ventilator-patient asynchrony and inspiratory WOB in intubated patients recovering from ARDS [146, 147]. Meeting the high demand at once during early inspiration lowers WOB [147]. When using mask NIV, the slope is typically set at 100–200 ms [148, 149].
- *Flow termination* should be achieved with the lowest expiratory trigger (lowest “cycling off”). First, with low compliance, the peak inspiratory flow is reached rapidly. Extremely early peak flow generated by the ventilator will terminate too rapidly the ventilator’s inspiratory flow sooner than the patient’s own inspiratory time, resulting in unmet demand and ventilator-patient asynchrony [59, 150]. Conversely, a long ventilator inspiratory time reduces asynchrony [2, 59] and increases Vt [135]. Second, a prolonged ventilator inspiratory time may activate the expiratory muscles to terminate the breath [150]. This leads to forced expiration and increased expiratory WOB [134]. Therefore, the inspiratory time should be neither too long (≤1s in acute distress [151]) nor too short. In the setting of invasive ventilation, cycling off is set from 1% of peak inspiratory flow [59] to 5% [134, 147]. In the setting of NIV, cycling off is 25–30% [2, 82].

#### 2. Helmet NIV

- *Standard setting*: Helmet NIV was recently reviewed [152]. Ventilatory flow>100 L.min^−1^ avoids CO_2_ rebreathing (CPAP systems: Series 500, Sea Long Medical System and CaStar, Starmed) [153]. As observed in the setting of HFN/VHFN, high flow increases the PEEP level. This may increase success when early severe diffuse ARDS is considered. By contrast, the helmet achieves less efficient pressurization and ventilator-patient synchrony. Nevertheless, new helmets are more comfortable and perform better [154]. Given the high compliance of the helmet, PS is modified [148]: fastest pressurization time≤50 ms (improved ventilator-patient synchrony), cycling off set at 50% of peak inspiratory flow down to 30% in case of double triggering [152], higher inspiratory assistance (+33–50%) and higher PEEP [71]. With this optimized synchrony, reduced RR, inspiratory effort, WOB, intubation rate and mortality are observed (intubation: mask: 61%; helmet: 18%; mortality: mask: 56%; helmet: 34%) [71].
- *Upfront helmet NIV*: Patients with the largest reduction in esophageal change do not require intubation [2]. Patients presenting with an inspiratory effort>10 cm H_2_O despite helmet NIV require intubation [82] (absence of improvement of labored breathing or of esophageal pressure changes [152], dyspnea, worsening oxygenation or ineffective coughing; mortality: 63%). The implication is that patients presenting with hypocapnia, vigorous inspiratory effort and severe lung injury require upfront helmet NIV and close observation to avoid delaying intubation, skipping HFN/VHFN/mask NIV [120]. Indeed, the absence of reduction of esophageal pressure changes (∆Pes) is associated with death under NIV [2]. Nevertheless, simultaneous to optimized physiological management (e.g., helmet), lowering the drive through a multimodal approach is required.

Patients presenting with a low inspiratory effort and small esophageal change on HFN require low PS, to avoid high transpulmonary pressure [82] during helmet NIV. When a high inspiratory effort and large negative esophageal change under HFN are observed, helmet NIV is superior to HFN (P/F≤200; shortest pressurization time, PEEP~10–12, PS~8–10 cm H_2_O; reduced dyspnea, intermediate discomfort) [82]. The reduction of inspiratory effort during helmet NIV was larger in patients with the largest inspiratory effort during HFN, linked to inflammation or deteriorating mechanics, but *not* to oxygenation [86]. Accordingly, patients presenting with low PaCO_2_<35 mm Hg benefit from helmet NIV, unlike patients with a high PaCO_2_>35 mm Hg [10].

Partial muscle relaxation [144] may represent an additional tool when the negative evolution of esophageal swings leads to helmet NIV combined to a multimodal approach, before a decision to intubate. In patients presenting with ARDS and a high Vt>8 mL.kg^−1^ a rocuronium infusion (5–37 mg over 6–60 min) was titrated to reduce the Vt (~9 to ~6 mL.kg^−1^, with increased PaCO_2_) and maintained for 2 h under conventional sedation (midazolam or propofol, sufentanil). Neurally adjusted ventilatory assist (NAVA) preserved diaphragmatic activity [144]. Such an approach may be useful in intubated or non-intubated patients under the care of anesthesia personnel with appropriate end tidal CO_2_, Vt, SaO_2_ monitoring. Although time-consuming, it may allow for the multimodal approach to achieve the temperature, agitation, systemic and microcirculation, kidney and metabolic goals under slow alpha-2 agonist sedation. Taken together, this suggests a 2 h window to improve the patient physiologically (HFN, NIV) [2, 72], then an additional 2 h using partial muscle relaxation [144], while running the multi-modal approach from admission onwards (Figure 1). Continued or increased labored breathing despite this full-fledged treatment implies intubation, and low PS under continued multimodal approach [9].

### Invasive ventilation, a rescue therapy

The sickest patients may benefit from immediate helmet NIV+multimodal approach. Within 2h, failed NIV leads to intubation+CMV+proning (*only* a “rescue” therapy) with continued multimodal approach. Early SB and upright position are used in the intubated patient as soon as the drive is normalized [9, 39,40,41]. Severe ARDS caused by e.g. peritonitis or acute pancreatitis necessitates upfront invasive ventilation until inflammation resolves. Indeed, all attempts delineated above may fail avoiding intubation, leading to effective invasive ventilation [100] or ECMO [100]. Less severe patients will undergo escalation under multimodal approach: HFN, then VHFN, and finally NIV (Figure 1). This approach may also apply in the setting of moderate septic shock [125].

Within the factors evoking hyperpnea and tachypnea (Equation 1), lung and systemic inflammation, metabolic acidosis and inadequate microcirculation are difficult to control. Many patients are managed with CMV either due to inappropriate NIV set up or inappropriate sedation with anesthetics/opioids, or extensive illness [20]. For example, full physiological support may coexist with high transpulmonary pressure (38 mm Hg), oedema, inflammation, and micro-emboli (PS=10, PEEP=15 cm H_2_O, ECMO to remove 77% VCO_2_, normalized pH, PaCO_2_, PaO_2_) [155]. Thus, when the drive exceeds the muscle capacity despite a multimodal approach, rigorous clinical criteria for intubation+CMV are needed.

## Pathophysiology and HFN/NIV merge in a multimodal approach

The multimodal approach (Figure 1) is common to HFN, VHFN, NIV and early SB following short term CMV+paralysis [9]. It relies on normalizing the respiratory drive: regardless of the ventilatory tool, the drive is normalized with Equation 1 as a checklist: (Vt, RR)=f(temperature, agitation, cardiac output, micro-circulation, inflammation, lung water-diuresis, systemic pH, PaCO_2_).

### 1. Fever control [156]

A baby lung allows only for baby O_2_ consumption (VO_2_) requirements. Thus, to reduce VO_2_, temperature is lowered to 36<θ<35°C i.e., the lowest temperature of human at night. In patients with reduced cardioventilatory reserve, VO_2_ is lowered [157] (~8–10% per °C [158] e.g., minus ~30% from 39.5 to 35.5°C). In ARDS patients, fever control is associated with improved survival [156]. Furthermore, in healthy volunteers, adrenaline infusion increases VO_2_ and Vt (respectively: +11; +17% [159]) and the inspiratory flow [159], unlike a reduced drive. As the ARDS patient is often septic and requires vasopressors, they further increase VO_2_.

By contrast, alpha-2 agonists lower a) the activation threshold of cold defense effectors (“set point”) [160,161,162] b) the temperature by >1°C in healthy volunteers [163] c) energy expenditure and VO_2_ by ~15–18% [163, 164] d) muscular tremor [165] and VO_2_, when baseline is high [166, 167]. Upon admission, paracetamol and external cooling are immediately followed by the administration of an alpha-2 agonist.

### 2. Cooperative sedation

Agitation independent of the ventilatory failure (such as anxiety, delirium, pain) is to be addressed. Dexmedetomidine or clonidine are administered as *first*-line sedatives to stringent quietness (−2<RASS<0; up to the “ceiling effect”: dexmedetomidine or clonidine: 1.5 or 2 µg.kg.h^−1^ respectively; *no* bolus administration; *starting with low doses and tirating slowly*; *fill them up* when hypovolemia is present; *open them up* if microcirculation is compromised [15,16,17]). The shorter half-life of dexmedetomidine facilitates nursing care (De Kock, personal communication). Alpha-2 agonists *combine* cardiac and vascular sympatholytic [168] and cardiac parasympathomimetic [169] actions, thus normalizing many factors within Equation 1. They evoke also sedation [170,171,172], slow wave sleep [173], normalize respiratory drive [174] with spontaneous breathing [39, 41], indifference to pain (“analgognosia” [175]) and to psychosocial or environmental stimuli (“imperturbability of mind”: ataraxia) [172], prevent delirium [176,177,178], reduce reactivity to noxious stimulus [179], especially in addict [180], young, combative patients. Importantly, alpha-2 agonists do not depress the respiratory generator (“generator”) [174]. The generator achieves adequate SB and NIV [181] without asynchrony and respiratory depression [163, 182]: a low Vt is observed with low or normal RR, according to temperature (35<θ<36°C). Indifference is achieved allowing for physiotherapy and continuous HFN/NIV [181, 183, 184] for days, *without* masking failure. Alpha-2 agonists lower the activity of the vasomotor center [185] and cardiac and vascular, arterial and venous, sympathetic activity, reduce the duration of CMV [13] and CCU stay [186], improve systolic [187] and diastolic [188] functions, normalize microcirculation [189], increase lactate clearance [190,191,192], lower noradrenaline requirement [193,194,195,196,197,198]. However, alone, alpha-2 agonists are useless. Only *combined* respiratory, ventilatory, circulatory, and autonomic interventions yield efficacy.

*Supplementation*: To achieve −2<RASS<0 and HFN/NIV for days, and given a ceiling effect [199], supplementation is sometimes required (“breakthrough”: haloperidol 2.5–10 mg i.v. bolus; infusion: haloperidol 50 mg/48 mL; 0.25 to 2 mL.h^−1^ [15,16,17]). Given the depression of the generator evoked by midazolam [174], propofol and opioids, we advise against anesthetics and opioids. They depress the drive, impose closer observation and complicate the management. In addition, hyperpnea may resume after curare or sedation withdrawal [200]. Enforcing sleep-wake cycle is crucial [173].

*Pain* management differ between medical and surgical patients, with medical patients typically requiring fewer analgesics compared to surgical patients. Opioid-free analgesia can be employed to avoid respiratory depression and SB suppression (e.g., ketamine 50 mg+nefopam 100 mg+tramadol 400 mg, 48 mL, 0.1–2 mL.h^−1^ [201]). Tramadol, being a weak opioid, has minimal respiratory depression effects. Cognition in elderly (nefopam) and acute kidney injury (tramadol) patients lead to rapidly lower the doses. The need for opioid-free analgesia typically decreases within 24–72h following administration of alpha-2 agonists.

*Cardio-respiratory coupling [44, 45] and sympathetic normalization*: First, there is a coordination between inspiratory phrenic and cervical sympathetic activity [202] (“respiratory-cardiovascular coupling”). Partial asphyxia evokes sympathetic activity throughout inspiration and expiration [202], in line with inadequate sympathetic hyperactivity in the setting of ARDS. Second, the interaction is also from the vasomotor center to the respiratory generator. Third, volume and vasopressors normalize the hypotension and the baroreflex-mediated sympathetic vascular hyperactivity. Following normalizing brain stem cardio-respiratory activity, attention can be focused on optimizing ventilatory mechanics, using appropriate tools (HFN, high PEEP-low PS, PEEP+CMV).

### 3. Normalized cardiac output (CO)

Alpha-2 agonists should not be used in cases of hypovolemia, sick sinus syndrome and atrio-ventricular block [15,16,17]. Positive pressure ventilation and PEEP require volume expansion to prevent hypotension and the need for vasopressors [203] as well as to avoid a pseudo-normalized intrapulmonary shunt [4].

- Adequate CO and adequate lung perfusion (Q) are necessary to normalize shunt. This also requires sufficient PEEP to achieve proper end-expiratory O_2_ diffusion (VA) [4]. Firstly, upfront normalization of CO enhances pulmonary flow (Q); second, high PEEP recruits ventilated alveoli (VA). Together, this normalizes the VA/Q distribution and improves oxygenation (patient 10 in [4]).
- Conversely, a pseudo-normalized shunt results from inadequate CO. First, high PEEP reduces CO, leading to decreased flow to unventilated alveoli, and an increased VA/Q ratio. Secondly, high PEEP increases the ventilation to unperfused alveoli, causing an increase in dead space [92]. As a resulted, despite an elevated PaO_2_, the shunt remains “pseudo-normalized” [4], as the skewed VA/Q distribution persists unchanged by PEEP itself, and the low VA/Q does not improve.

To achieve adequate VA/Q, first, iterative echocardiography monitors the ventilation-induced changes in vena cava diameter, the right ventricular dilation, the mitral and aortic flows, the left ventricular [LV] contractility and the presence of foramen ovale (present in ~20% of the patients [204]). Various tools as volume, vasopressor, inotrope, pulmonary vasodilator are used to achieve adequate CO, mixed venous saturation, CO_2_ gap, pH and lactate. Additionally, the combination of PEEP and SB acts synergistically. SB evokes diaphragmatic compression of the hepatosplanchnic blood [205] enhancing venous return, while PEEP decreases LV afterload [206]. Second, in addition to arterial and venous gases, impedance tomography or lung echography may assist in observing adequate VA.

*Sympathetic normalization and improved microcirculation*: First, the heightened vascular sympathetic activity is associated with high lactate [207]. The alpha-2 agonists normalize the sympathetic activity, the microcirculation [189] and the lactate concentration [189,190,191,192]. Systemic and regional metabolic acidosis is normalized within ~3–6 h, lowering peripheral inflammation and respiratory drive. Lastly, acute kidney injury and associated metabolic acidosis are managed with renal replacement therapy. In summary, *counterintuitively*, the treatment approach for ARDS prioritizes circulatory intervention [4, 46, 203, 208, 209] followed by ventilatory strategy.

### 4. Inflammation

Patients have transitioned from young trauma patients with preserved immune system at baseline and heading into severe delayed injury-acquired immunodeficiency [210], to elderly patients presenting with chronic baseline heightened inflammation, such as those with COVID-ARDS. Acute inflammation can result from conditions like sepsis, emphasizing the importance of early source control, or systemic acidosis, or impaired ventilatory mechanics (SILI or VILI).

- Direct immuno-modulation can be targeted (e.g., anti-IL-6) or non-targeted (e.g., steroids). Both address the non-mechanical inflammation caused by the disease (e.g., steroids and SARS-CoV2 [211]) or the syndrome (e.g., systemic sepsis). In addition, steroids may address the inflammation caused by atelectrauma and SILI.
- Indirect immuno-modulation: alpha-2 agonists present *indirect* systemic anti-inflammatory effects, a facet too often overlooked [195, 212,213,214,215,216,217,218,219]. They normalize heightened sympathetic hyperactivity, and upregulate beta-adrenergic receptors on lymphocytes [220]. This mechanism may extend to normalizing the functioning of all adrenergic receptors on all immune-competent cells. This may alleviate immuno-paralysis.
- Mechanical inflammation and SILI: Reduction of esophageal pressure changes is co-related to Vt reduction and radiologic improvement, respectively after 12 and 24 h [2]. Therefore, a normalized drive normalizes the WOB and suppresses SILI [5], early on.

### 5. Lung water

Reducing lung water is crucial [221, 222] when inflammation [223] play a significant role, such as in high permeability edema or large negative esophageal changes. Once CO is normalized, volume infusion should be minimized. Indeed, in the setting of SB, low Vt and compliance [222], a ~10–15% CO increase to passive leg raising does *not* necessarily indicate the need for further hydration. To minimize lung water, the overall response is considered, at variance with BP or CO themselves: mottling, capillary refill time [224], urine output, venous SO_2_, lactate, CO_2_ gap, vena cava ventilatory changes.

Additionally, a) SB facilitates better lymphatic drainage compared to CMV [225] b) sympathetic blockade reduces pulmonary vein pressure and lung edema [226] c) alpha-2 agonists evoke diuresis through an anti-ADH effect [227]. The issue is not anymore the total volume of fluids administered during early resuscitation or the first day on admission, but the overall *balance* of fluids and weight achieved after 24, 48, 72 h d) following organophosphate poisoning, clonidine suppresses capillary filtration, thus pulmonary edema [228]. f) following lung contusion, clonidine improves inflammation [229].

### 6. CO_2_

Hypocapnia is an ominous sign in the setting of early ARDS [72]. PaCO_2_ is lower when NIV failure occurs [2, 72], *irrespective* of P/F (>200 [72]; 101–170 [2]). This hypocapnia is close to the apneic threshold (healthy volunteer: ~30–35 mm Hg; NIV failure: 32 mm Hg [72]; high inflammatory status e.g. COVID: ≤30 mm Hg). This suggests switching early to helmet NIV [82, 120].

The striking observation is the occurrence of hyperpnea *and* hypocapnia, below the apneic threshold [230, 231]. Indeed, the threshold is overridden by systemic acidosis or central nervous system inflammation or stimulation of lung receptors [42, 43]. Athletes enduring Vt≥3 L, minute ventilation>160 L.min^−1^ and esophageal pressure changes≥60 cm H_2_O for hours [77, 119, 232] suggest that increased Vt *per se* is not detrimental, but rather inflammation plays a significant role. Could this be a consequence of pH, PaCO_2_ or metabolic or cortical excitatory inputs onto the respiratory generator? If so, the respiratory generator should be made refractory to psychosocial stimuli generated by the CCU environment *without* suppressing respiratory genesis itself [174], and SB. This approach aims to alleviate increased respiratory drive and sympathetic hyperactivity, without resorting to general anesthesia and paralysis.

In COVID-ARDS, under paralysis+CMV, micro- or macrothrombi leads to high dead space, hypercapnia and a high respiratory drive: a low normal temperature (35–36°C) will help normalizing the VCO_2_ and hypercapnic drive, allowing for SB, under HFN/NIV.

### 7. O_2_

Hypoxemia, silent without or with overt failure, requires immediate treatment. Nevertheless, alleviating hypoxemia is not the ultimate objective:

- Improved oxygenation and reduced mortality are unrelated [233]. Thus, low SaO_2_ alone is not an indication for intubation [21]; rather labored breathing and impending/overt failure are.
- In rats, inflammation increases in response to acute hypoxia, *independent* of the degree of hypoxemia [234]. To address increased WOB, correction of hypoxemia *per se* is not the immediate goal.
- In late-stage ARDS, hypoxemia is associated with increased RR and reversed by high FiO_2_ [78]. This holds true in early ARDS: in non-intubated non-paralyzed patients, the hypoxic drive should be suppressed by combining high FiO_2_ with the highest PEEP achievable with HFN/NIV. Permissive hypoxemia is avoided to lower Vt and RR, ideally without hyperoxemia (SaO_2_≥92–100%: roughly the flat portion of the dissociation curve).
- Hypoxemia act as a *transient* stimulus, briefly enhancing the ventilatory response to hypercapnia or metabolic acidosis [74, 235] (“*hypoxic ventilatory decline*”). Given the hypocapnia observed in early ARDS [2, 117], the relevant stimulus is not hypoxemia but systemic acidosis or the metaboreflex (“originating in skeletal muscle activated when blood flow to contracting muscles is insufficient to allow both O_2_ delivery and metabolite washout” [3]). Furthermore, a) age and diabetes blunt the response to hypoxemia [235]. b) in the setting of early ARDS, silent hypoxemia may occur without dyspnea [73,74,75, 235].

Rather than simultaneously lowering the FiO_2_ and the PEEP [97], they are adjusted *sequentially* [37, 41, 236].

- *Lowering FiO_2_*: a) Absorption atelectasis [237] necessitates minimizing the duration of FiO_2_=1 administration. b) The highest possible O_2_ flow sets PEEP as high as possible given the leaks observed under HFN/NIV. Hypoxemia improves in most patients with a moderate PEEP (5–15 cm H_2_O) achieved with HFN/VHFN [121, 122]. Subsequently, with the highest achievable PEEP and a successful response to the multimodal approach, FiO_2_ is gradually reduced from 1 to 0.4.
- *Lowering PEEP*: Under FiO_2_=0.4 and *constant* SaO_2_≥96%, PEEP is gradually reduced from ~15 [122] to ~5 cm H_2_O [121], by flow reduction. As the mechanical properties of the lung improve slowly [238, 239], achieving a SaO_2_≥96% requires patience, in contrast to the rapid effects seen with recruitment maneuvers [95]. If deterioration occurs again, it suggests i) investigating underlying causes such as sepsis, coronary occlusion, delirium ii) implementing helmet NIV in cases of persistent or worsening labored breathing iii) revisiting the entire multimodal approach.

### 8. Position

The supine position worsens sick human (reduced FRC, increased abdominal pressure with atelectasis next to the diaphragm) [237]. Thus, the upright position presents some rationale to improve oxygenation [7]. Nevertheless, the head up position may worsen compliance and driving pressure in late ARDS (“paradoxical” positioning [240]). Furthermore, the rationale for extended upright intervals in a healthy human does not automatically transfer to a sick biped. To our knowledge, upright has not been documented in the setting of COVID-ARDS. As VHFN/NIV may evoke gastric dilation [2], the intraabdominal pressure should be reduced early (gastric and bladder catheters, enhanced intestinal motility).

## Conclusion

This multimodal approach bases itself on progress in the pathophysiology of ARDS [2, 6, 8, 42, 43, 72]. This synthesis of autonomic, respiratory, circulatory and ventilatory physiological advances combines with technological advances to avoid intubation, unless “absolutely necessary” [21]. Would this allow to reap “*the far-reaching benefits of spontaneous yet highly supported ventilation in an awake, animated patient over invasive mechanical ventilation via endotracheal tube*” [71]? A prospective randomized pilot trial, then a larger trial are required to ascertain the working hypotheses delineated above.

## Abbreviations and glossary

ARDS: acute respiratory distress syndrome
Analgognosia: indifference to pain.
Ataraxia: “imperturbability of mind” (Epicurus) [1]
BP: blood pressure
bpm: breath per minute
C-ARDS: SARS-CoV2 evoked acute respiratory distress syndrome
CCU: critical care unit
CMV: controlled mandatory ventilation
CO: cardiac output
COPD: chronic obstructive pulmonary disease
Cstat: static compliance: Vt/Plat-PEEP, with an inspiratory pause (no flow).
Cdyn: dynamic compliance: Vt/PIP-PEEP, without inspiratory pause (with flow); surrogate: Vte/∆PL [2].
CPAP: continuous positive airway pressure
Dependent lung: in standing human, basal, non-aerated lung
DP: driving pressure
∆Pes: esophageal pressure drop
∆PL: tidal change in dynamic transpulmonary pressure
Failure: ventilatory failure
FRC: functional residual capacity
GA: general anesthesia
Generator: respiratory generator located in the lower brain stem setting the respiratory rhythm
HFN: O_2_ high flow nasal
HR: heart rate
Inspiratory effort: quantified by negative changes in esophageal pressure
Labored breathing: continued or intensified labored breathing, heading to ventilatory failure
Metaboreflex: “originating in skeletal muscle activated when blood flow to contracting muscles is insufficient to allow both O_2_ delivery and metabolite washout” [3]
NIV: non-invasive ventilation
Non-dependent lung: in standing human, apical, aerated lung
PEEP: positive end-expiratory pressure
Pendelluft: intrapulmonary gas redistribution from nondependent (better aerated) to dependent lung without Vt change
Pes: surrogate of transpulmonary pressure (alveolar pressure minus pleural pressure)
P/F: PaO_2_/FiO_2_
Pplat: pressure measured during brief end-inspiratory hold
PS: pressure support, inspiratory assistance
P-V curve: pressure-volume curve
RASS: Richmond agitation sedation scale
RR: respiratory rate Respiratory physiology refers to the brain stem respiratory generator and phrenic activity
ROX index: (SaO_2_/FiO_2_)/respiratory rate; sicker patients require more oxygen and a higher respiratory rate.
SB: spontaneous breathing, spontaneous ventilation
SILI: patient’s self-induced lung injury
SO_2_: oxygen saturation
Shunt: perfusion of non-aerated alveoli (low or zero VA/Q [4]); Qs/Qt=(CcO_2_ − CvO_2_)/(CcO_2_ − CaO_2_)
Strain: lung deformation [5], tidal volume, tidal volume/end expiratory lung volume [6]
Stress: transpulmonary pressure [5, 6], driving pressure
Transpulmonary pressure: transmural pressure between the inside (alveolus) and outside (pleura-esophagus) of the cavity
Upright position: reverse Trendelenburg, head-up +60°, legs down: −45° [7]
VA/Q: ventilation/perfusion ratio
Venous admixture: intrapulmonary shunt+VA/Q mismatch (low VA/Q areas) [8] Ventilatory physiology refers to lung and chest wall mechanics
VHFN: O_2_ very high flow nasal
VILI: ventilator-induced lung injury
VO_2_: oxygen consumption
Vt: tidal volume
WOB: work of breathing

## Appendix

This overview outlines a step-by-step escalation based on the severity of ARDS (HFN, VHFN, mask NIV, helmet NIV, invasive ventilation). Despite some research addressing these questions, there remains limited knowledge about SB compared to controlled mechanical ventilation with and without paralysis.

### A. Research questions

As of now, there is a lack of data regarding the measurement of plateau pressure (Pplat) during a brief inspiratory hold in patients receiving non-invasive ventilation (NIV) without an endotracheal tube [137]. Lung mechanics must be re-evaluated specifically in the context of SB, both with and without intubation. This includes re-adressing in the setting of NIV all the parameters used for PS in the setting of invasive ventilation.

Is NIV success related to high PEEP and suppressed solid-like behavior and atelectrauma (reduced inspiratory effort consequence of improved mechanics (PEEP))? is NIV success related to adequate inspiratory assistance and unloading the muscles? [36, 86, 117, 143, 242]; are sick patients with a high drive homogeneous to less severe patients?

Delineate physiologically each factor (temperature, agitation, cardiac output, blood pressure, pH, PaCO_2_) involved in labored breathing, hyperpnea and large changes in esophageal pressure before [2, 72] or after [243, 244] intubation? If mortality is lower [39, 41], what is the mechanism: fever control [119, 245]? normalized sympathetic activity? improved microcirculation? lowered lactate? lowered inflammation? extended tolerance to NIV? minimized leaks and higher PEEP? absence of conventional sedation?

A normalized drive normalizes the WOB and suppresses SILI [5], early. Causality should be clarified: i) SILI is the limiting factor in early ARDS [48, 246] ii) increased WOB then overt failure is the limiting factor [20, 21] iii) both.

Readdress the RV and LV performance in the setting of SB vs CMV+GA?

Quantify the WOB vs. SaO_2_, a putative reduction of esophageal pressure changes and reduction of rate of intubation using VHFN>120 L.min^−1^?

Do pharmacological tools exist to deactivate lung receptors (mechano-, A∂ and C, J) and minimize lung inflammation?

Adressing Vt in the setting of severe diffuse ARDS (2,4, 6, 8, etc. mL.kg^−1^) with or without veno-venous extracorporeal membrane oxygenation (ECMO), with or without low normal temperature (35–36°C)?

Document the effect of supine vs. proning vs. lateral+prone+lateral repositioning (physiology, CT scan, epidemiology)?

Document the effect of passive hyperventilation without sedation [114] on outcome?

Compare and combine tools to individualize PEEP : esophageal balloon vs impedance tomography vs lung echography?

In addition to the nasal prong, insertion of up to 2 prongs through the mouth would achieve O_2_ flow ~120–180 L.min^−1^. Do adequate numbers (SaO_2_) translate into improved outcome?

Readdress pressure support vs airway pressure release ventilation.

Assess partial muscle relaxation [144] in the setting of high Vt and helmet NIV on outcome ? does partial muscle relaxation allows for extended observation with improved outcome?

VHFN appears poorly tolerated, after 20 min [123]. The reason is unclear : poor humidification? high expiratory resistance and expiratory WOB [123]?

Heightened sympathetic hyperactivity downregulates beta-adrenergic receptors on lymphocytes; normalized sympathetic activity upregulates beta adrenergic receptors [220]. Would this improve all adrenergic receptors on all immuno-competent cells? Would this lead to improved immuno-paralysis?

### B. Randomized clinical trial

The PICO (patient, intervention, comparison, outcome) question is: does combining new pathophysiological information reduce intubation+controlled mandatory ventilation (CMV)+paralysis+deep sedation and improve outcome in the setting of early ARDS, manpower, bed and anesthetics shortage and mass influx of patients with baseline chronic inflammation (e.g., COVID-ARDS)?

Following a pilot trial, a prospective trial should randomize patients, with outcome as the primary endpoint:

1. Physiology: Patients presenting with high Vt and or large esophageal pressure swings or low P/F to a 2 h helmet trial, followed if negative with partial muscle relaxation [144] to lower Vt~6 mL.kg^−1^.
2. Multimodal approach (physiology + pharmacology): Patients presenting with high Vt and or large esophageal pressure swings or low P/F to a 2 h helmet trial, followed if negative with partial muscle relaxation to lower Vt~6 mL.kg^−1^, combined to a multimodal approach as delineated in text (figure 1).
3. State-of-the-art: Patients presenting with high Vt and or large esophageal pressure swings or low P/F to intubation, controlled mechanical ventilation (driving pressure<14 cm H_2_O, PEEP according to NIH table [97] or better to esophageal balloon [99, 100], general anesthesia+paralysis+proning as the state of the art in severe ARDS.
